# Psychopathy is a key to integrating dimensional personality disorder constructs and psychotherapy: toward a reformulation of personality and its disruption

**DOI:** 10.3389/fpsyt.2026.1937903

**Published:** 2026-10-22

**Authors:** Aina Gullhaugen

**Affiliations:** St. Olav’s University Hospital, Nidaros District Psychiatric Center (DPS), Trondheim, Norway

**Keywords:** psychopathy, personality disorder, personality, dimensional, integrating

## Abstract

While the conceptualization of personality disorder (PD) moves toward a dimensional approach, the broader adoption within clinical practice appears slow. The lack of a unifying framework in diagnostic classifications might explain this issue. Here, psychopathy, arguably the most pervasive and treatment-resistant PD variant, is presented as a key to integrating dimensional PD constructs and psychotherapy. The Dynamic Model of Psychopathy Assessment and Treatment Framework (DMP-ATF) provides a coalescing structure that, by virtue of its characterization of pivotal developmental psychological processes, hypothetically transcends variations in population, age, and clinical severity, to situate PD within a universal context. The redefined and integrated psychopathy construct and its associated framework represent a meta-theoretical approach that can unify therapeutic models, techniques, and principles to advance psychotherapy integration based on dimensional PD constructs, while placing personality and its psychopathology within a common structure. A system for converting externalizing behaviors into self-conscious emotions and needs, while managing risk, is additionally provided. While these proposals are compatible with contemporary biopsychosocial and treatment-oriented PD models and a trending treatment optimism, they await empirical validation.

## 1 Introduction: the lack of a unifying framework in diagnostic classifications

While the conceptualization of personality disorder (PD) moves toward a dimensional approach, the broader adoption within clinical practice appears slow (1, 2). The lack of a unifying framework in diagnostic classifications might explain this issue (3, 4), as neither the descriptive list of trait domains based on conceptualizations of personality (negative affectivity; detachment; antagonism; disinhibition; psychoticism) nor the level of impairment in self-interpersonal functioning (identity; self-direction; empathy; intimacy) of the DSM-5 Alternative Model for PD (AMPD) (5) or their ICD-11 equivalents (6) sufficiently explain what PD “is” (essence; coherence) and “does” (function; purpose) (7), with implications for treatment. Concurrent dimensional and integrative taxonomies like the HiTOP (8), externalizing-spectrum models (9), five-factor approaches (10), and interpersonal models (11) only emphasizes this point. This situation parallels the confusion regarding the construct of psychopathy (12) and its treatment (13). Here, psychopathy, arguably the most pervasive and treatment-resistant PD variant is, nonetheless, presented as a key to integrating dimensional PD constructs and psychotherapy and, in this, providing an opportunity to potentially unite the field.

### 1.1 Psychopathy as a diagnostic endpoint for personality pathology

The current clinical construct of psychopathy originated in Hervey Cleckley’s book The mask of sanity: An attempt to clarify some issues about the so-called psychopathic personality (14). Sixteen characteristics were derived from the several cases he encountered as a psychiatrist. Traits such as a grandiose sense of self-worth, lack of remorse or shame, unresponsiveness in interpersonal relations, and failure to follow any life plan were later empirically validated and adapted to a criminal population by psychologist Robert Hare. Hare’s Psychopathy Checklist-Revised (PCL-R) is the most researched psychopathy model, including 20 items (of which two fail to be^1^) grouped into 2–4 factors (interpersonal; affective; lifestyle; antisocial) without a theoretical foundation (15). Like the PCL-R, the Triarchic Psychopathy Model (with blends of phenotypes related to boldness, meanness, and disinhibition) (16) and the Comprehensive Assessment of Psychopathic Personality (CAPP; with attachment, behavioral, cognitive, dominance, emotional, and self domains) (17) represent little actual innovation in terms of disputing Cleckley’s features^2^, but by embracing developmental plasticity and a capacity for change they nevertheless challenged the biodeterministic paradigm that for long permeated the field (18) as a stricter version of the difficult to treat-attitude and the stigma surrounding PDs in general, that is about to change (e.g., due to contact-based interventions) (19).

Diagnostically, psychopathy is associated with antisocial PD (5), which is probably a misleading conceptualization as individuals who meet the PCL-R criteria can be diagnosed with PDs from all diagnostic clusters (20, 21), although cluster B disorders are most often reported (22, 23). In fact, Khalifa and Howard (24) emphasize that “rather than representing a homogeneous clinical entity, psychopathy in its various guises might run like a seam through, and across, the different categories of PD described in DSM-5” (p. 863). Thus, unlike the specific PDs associated with one of the four quadrants (25) of Leary’s (26) Interpersonal circumplex (IC) with orthogonal dimensions of power (dominance-submission; vertical axis) and affiliation (hostility-affection; horizontal axis), psychopathic dispositions might, similar to borderline PD (3, 27), be linked to all IC quadrants.

Reports of negative correlations between the PCL-R and cluster C dependent and avoidant PDs (20) can be explained by the fact that, although the PCL-R includes the intense interest and engagement in others (cf. the items that do not load on any factor) that we find in a broader and more persistent or rigid variant in dependent PD, experts fail to recognize its conceptual significance; affective and interpersonal detachment is the foundational premise (Factor 1). And although the PCL-R includes a nonassertiveness (irresponsibility; parasitic lifestyle; lack of realistic long-term goals) that can be related to dependent and avoidant PDs, the lifestyle (i.e., impulsive) and antisocial behavior is the propensity at issue (Factor 2). *If* the interpersonal dispositions of psychopathic individuals can be positively associated with all IC quadrants (hostile, affiliative, dominant *and* submissive tendencies; heterogeneity in terms of dispositional differences), a dimension of self- and affect consciousness (defined as reflection on or awareness of intrinsic experiences and states, value and uniqueness) can explain the temporal heterogeneity in relational features found in borderline (28) and psychopathic (29–31)^3^ PDs *and* separate borderline (negative self-image; emotionally unstable; self-harm) from psychopathic (grandiose self-image; emotionally callous; harm to others)^4^ disorder. Thus, rather than being a distinct variant of antisocial PD, *psychopathy could represent a diagnostic endpoint for PD* characterized by a lack of affiliation, power *and* self- and affect consciousness, reflecting an extreme disruption or the most self-protective manifestation of the three dimensions by which all PD variants can potentially be explained. This can be tested by comparing PD variants on measures of affiliation, power, and self- and affect consciousness. **Table 1** displays research questions and hypotheses for future empirical validation, organized by order of appearance in this article.

**Table 1:** Research questions and hypotheses for empirical validation, organized by order of appearance in the article.

Research questions and hypotheses for empirical validation organized by order of appearance in the article
1. Introduction: The lack of a unifying framework in diagnostic classifications 1.1. Psychopathy as a diagnostic endpoint for personality pathology RQ_1_: Does psychopathy represent a diagnostic endpoint for PD characterized by a lack of affiliation, power, and self- and affect consciousness, reflecting an extreme disruption or the most self-protective manifestation of the three dimensions by which all PD variants can possibly be explained? H_1_: Psychopathy represents the most disruptive or self-protective manifestation of the dimensions, as evidenced by grandiose, detached, and dominant tendencies and less frequent shifts on the IC relative to borderline PD, as shame operates unconsciously. 1.2. The compatibility and utility of a dimension of self- and affect consciousness RQ_1_: Does the self- and affect consciousness dimension serve to simplify the diagnostic PD nomenclature? H_1_: The DSM-5 AMPD and the ICD-11 functioning impairments can be organized according to the self- and affect consciousness dimension and Leary’s IC affiliation and power dimensions. RQ_2_: Can personality be conceptualized more accurately as a general trajectory of personological maturation hypothetically measured as individual balance (not rigidity or shifts between extreme states) and thus variance on the three dimensions (self- and affect consciousness; affiliation; power)? H_2_: Individual balance and thus variance on the three dimensions (self- and affect consciousness; affiliation; power) serves as an index or metric of personological maturation, i.e. personality. RQ_3_: Can PD, unlike other mental disorders, be addressed as lacks in integrity, attachment, and autonomy? H_3_: PD is uniquely distinguished from other mental disorders by a structural imbalance within integrity, attachment, and autonomy, manifested as rigidity and shifts between extreme states, whereas other conditions maintain a degree of individual balance. RQ_4_: Can the DMP counter the lack of an integrated understanding that has been identified as a major cause of the treatment difficulties associated with the disorder, by explicating a dynamic interplay of affective-interpersonal, conscious-unconscious states that transcends populations, age, and severity levels as parts of developmental psychological processes which applies to all people? H_4_: The DMP counters the lack of an integrated understanding by explicating a dynamic interplay of states transcending populations as parts of universal developmental processes. 2. Psychopathy is a key to integrating dimensional PD constructs and psychotherapy 2.1. The Dynamic Model of Psychopathy Assessment and Treatment Framework (DMP-ATF) 2.1.1. DMP Assessment RQ_1_: Does the DMP make sense of the PCL-R’s atheoretical cut-off for severe psychopathy by explicating how externalized or unconscious shame, evidence by anxiety-reducing compensation or self-enhancement (verbal and non-verbal communication of an inflated sense of superiority, self-importance, and uniqueness that is not based on actual capability), will fail to trigger empathy and to inhibit ruthless behavior? H_1_: Externalized or unconscious shame, as evidenced by an anxiety-reducing compensation or self-enhancement, will fail to trigger empathy and to inhibit ruthless behavior. RQ_2_: Can the DMP-ATF personalize treatment regardless of PD configuration and the distance to the treatment goals? H_2_: Individual placement on the DMP dimensions personalizes treatment regardless of PD configuration and the distance to the treatment goals. 2.1.2. DMP Treatment RQ_1_: Does low emotional awareness equal high risk of goal-directed behavior (using others to satisfy needs) and the instrumental (not preceded by an affective reaction) violence that is characteristic of psychopathy? H_1_: Diminished emotional awareness elevates the risk for the goal directed behavior and the instrumental violence characteristically observed in psychopathy. RQ_2_: Can treatment within the DMP-ATF be a high precision tool that may significantly reduce treatment duration and the risk of relapse? H_2_: The thorough and continued assessment associated with developmental psychological milestones and the focus on states and emotional awareness, reduces treatment duration and the risk of relapse, which makes the DMP-ATF a high precision tool.

### 1.2 The compatibility and the utility of a dimension of self- and affect consciousness

The potential need for adding “new spatial dimensions to the organization of personality” (p. 64) was anticipated by Leary (26), and the proposed self- and affect consciousness dimension is compatible with the DSM-5 AMPD and the ICD-11 PD sections positing identity problems and self-functioning impairment^5^ as transdiagnostic factors across personality pathology. In extension, deficits in self- and affect consciousness would explain the lack of predominantly affective, not cognitive, empathy that is a hallmark of psychopathy (associated with the callousness component of the construct), whereas a more unbiased affective functioning (linked to antisociality) might explain lacks in cognitive empathy (35)^6^. This suggests that the empathy functioning impairment of the DSM-5 AMPD and the ICD-11 focusing on perspective taking might be redundant. The remaining DSM-5 and ICD-11 functioning impairments can be organized according to the self- and affect consciousness dimension (ICD-11 self-worth, accuracy of self-view, and perspective-taking) or Leary’s IC community and agency dimensions (AMPD intimacy = IC community; self-direction = IC agency; ICD-11 self-direction and conflict management = IC agency, ICD-11 close relationships = IC community). Altogether, a hypothesized benefit of the self- and affect consciousness dimension is that *it may serve to simplify the diagnostic nomenclature* (**Table 1**), which is needed according to experts (37).

The IC was one of several frameworks that were considered for inclusion in the revision of the DSM-IV (38) that lost to the trait approach. Research however indicates that the AMPD Criterion A (level of impairment in self-other functioning) predicts the presence of PD in general, while the personality based trait domains (stylistic expressions of the incapacity in Criterion B) lose incremental predictive value after Criterion A is included (39, 40). Experts, however, still agree on the importance of integrating personality and psychopathology within a common structure (34). Thus, *if* the current concept of personality cannot satisfactorily explain PD, the dimensions that are required to predict or describe PD (tentatively; self- and affect consciousness, affiliation, and power) can be utilized to clarify personality, a third potential utility of the self- and affect consciousness dimension.

The largely unipolar PD models where opposing poles represent under- and overcontrolled aspects of each personality disturbance, with a middle response reflecting normal functioning, would assume the bipolar nature of normal personality (2, 38). A healthy personality would thus involve balance^7^ not rigidity in or shifts between extreme states^8^ of self- and affect consciousness, affiliation, and power, which can explain what PD “is” (lacks in integrity, attachment, and autonomy) and “does” (reaching for milestones^9^). Thus, rather than lists of more or less related trait domains (modern usage of the personality construct), personality might be conceptualized more accurately as “a general trajectory of personological maturation”^10^ ^11^ (43) hypothetically measured as *individual balance and thus variance on the three dimensions* (**Table 1**). This is a comprehensive and integrative model incorporating functional definitions of personality and psychopathology that is compatible with the next horizon for personality and psychopathology research as it is proposed by Wright and Hopwood (34). Zavlis et al.’s (7) generative model of PD formalizing personality pathology as probabilistic, mentalizing, hierarchical, and dynamic runs along these lines, but is less cohesive (the four key properties lack clear integration) and specific (mechanisms remain to be fully delineated).

The idea of psychopathy as a diagnostic endpoint for PD is, in brief, in keeping with psychopathy being the first PD to be recognized in psychiatry, referring to a variety of PDs as extreme variants of normal personality (23). It is also well-matched with the reformulated PD section of the ICD-11 (6), where the separate diagnoses are reduced to one type (mild, moderate or severe) whose configuration should be based on five trait domains (negative affect; dissocial; disinhibition; anankastic; detachment), all of which are relevant to psychopathy (as is the AMPD’s focus on clinical significant problems in functioning and pathological traits) (5). The comorbidity between psychopathy (PCL-R) and the distinct PDs (rigid coping, e.g. the cold tendencies of antisocial PD *or* the overly nurturant tendencies of histrionic PD) (25) further signals that while these individuals are psychopathic, the way psychopathy is expressed tends to shift (inconsistent coping). This is compatible with the borderline pattern (pervasive instability in relationships, self-image, emotions, marked impulsivity) included in the ICD-11 and reports of such personality structure, described by Kernberg (44), in borderline PD (45) and in PCL-R high-scorers (29–31). The idea of psychopathy as a diagnostic endpoint for PD however challenges Kernberg’s perception of psychopathy as an *antisocial* PD proper (46) belonging to the high end of a spectrum of pathological narcissism, as it can be argued that all PDs are narcissistically inclined (through their compensation) and thus involve ruthless while not necessarily objectively antisocial behavior (like the pronounced reassurance-seeking behavior and interpersonal dependence characteristic of individuals with dependent PD will clash with the more balanced desire for community and autonomy found in the general population). In psychopathy, pathological narcissism is indeed central, pointing in the direction of psychopathy being the complete form of PD *per se* or PD coming full circle. Lastly, the hypothesis of psychopathy as a diagnostic endpoint for PD builds on an extension of and is thus consistent with interpersonal models (11). The 5 factor approach (10) potentially accommodate such redefinition, which is also the case within the HiTOP (8). Externalization models (9) will, however, fail to provide an exhaustive account.

The utility of identifying a diagnostic endpoint as an extreme state of PD or relative point of stasis in an otherwise dynamic elaboration of personality does not intervene with the moving away from categorical descriptions to the idea that the degree of PD is more central than the type, but may define the boundaries of movement (variations can potentially be explained by three dimensions) and *how PDs differ from other mental disorders (lacks in integrity, attachment, and autonomy;* **Table 1**); a forth potential benefit of the self- and affect consciousness dimension. Nor does it denote a psychotic eroded self-other demarcation, but an extreme narcissistic investment and denial of the status of others as subjects in their own right (PDs as interpersonal disorders) (7, 11). Such redefinition and integration of psychopathy as a construct makes it possible to dispose of it due to stigma. By keeping it, its etymological connotation “a suffering mind” will remind us about the struggles that individuals with psychopathy face without openly stating it. Cleckley (14) dismissed the attempt to grasp a conceivable process behind this mask, as it could not be reached through verbal exploration and direct examination. By adding a dimension of self- and affect consciousness to the IC, new research areas (47) and methods (48) are inevitable, targeting emotional awareness in a relational context.

In sum, the first part of this article establishes how psychopathy might be central to integrating dimensional PD constructs, a step toward a more streamlined and unifying framework in diagnostic classifications. By adding a dimension of self- and affect consciousness to Leary’s IC, psychopathy hypothetically achieves an independent status and can be reintegrated among the other (former) PD types as the most severe variant, which has conceptual significance in a dimensional framework as the levels of impairment must be clarified. Further, callous grandiosity can be explained as a failure to maintain a stable and healthy sense of self, inferring a vulnerability in psychopathy that can reduce the stigma and offer a productive basis for further inquiry. Personality was redefined, based on the three dimensions that are hypothetically required to explain PD, and PD can be distinguished from other mental disorders by addressing concerns related to self- and affect consciousness, affiliation, and power. New assessment and treatment approaches are inevitable, and clear-cut treatment goals (cf. section 2.1.2) come with the potential of increasing precision and treatment outcomes. Overall, this suggests a fundamental change in the basic concepts and practices in this field. Traditional theoretical frameworks (static traits) can be replaced with one that better explains the observed phenomena (dynamic states) and addresses limitations of the previous models (e.g., the PCL-R items that do not load on any factor) (15), which are the prerequisites for a paradigm shift.

The Dynamic Model of Psychopathy (DMP; **Figure 1**) (49) proceeds from this way of thinking and might counter the lack of an integrated understanding that has been identified as a major cause of the treatment trouble associated with the disorder (13), by explicating a dynamic interplay of affective-interpersonal, conscious-unconscious states *hypothetically (***Table 1***) transcending populations, age, and severity levels, as parts of developmental psychological processes that applies to all people* (sex and gender, culture, comorbidity, etc., might also influence the final expression). This fits a transdiagnostic approach that centers on modes or “state-like manifestations of personality that function as cohesive organizational units, characterized by specific profiles of affects, behaviors, cognitions, and desires that tend to be co-activated” (p. 238) (50). Next, the DMP is introduced before its assessment and treatment framework is presented, which is potentially relevant to all PD.

**Figure 1:**
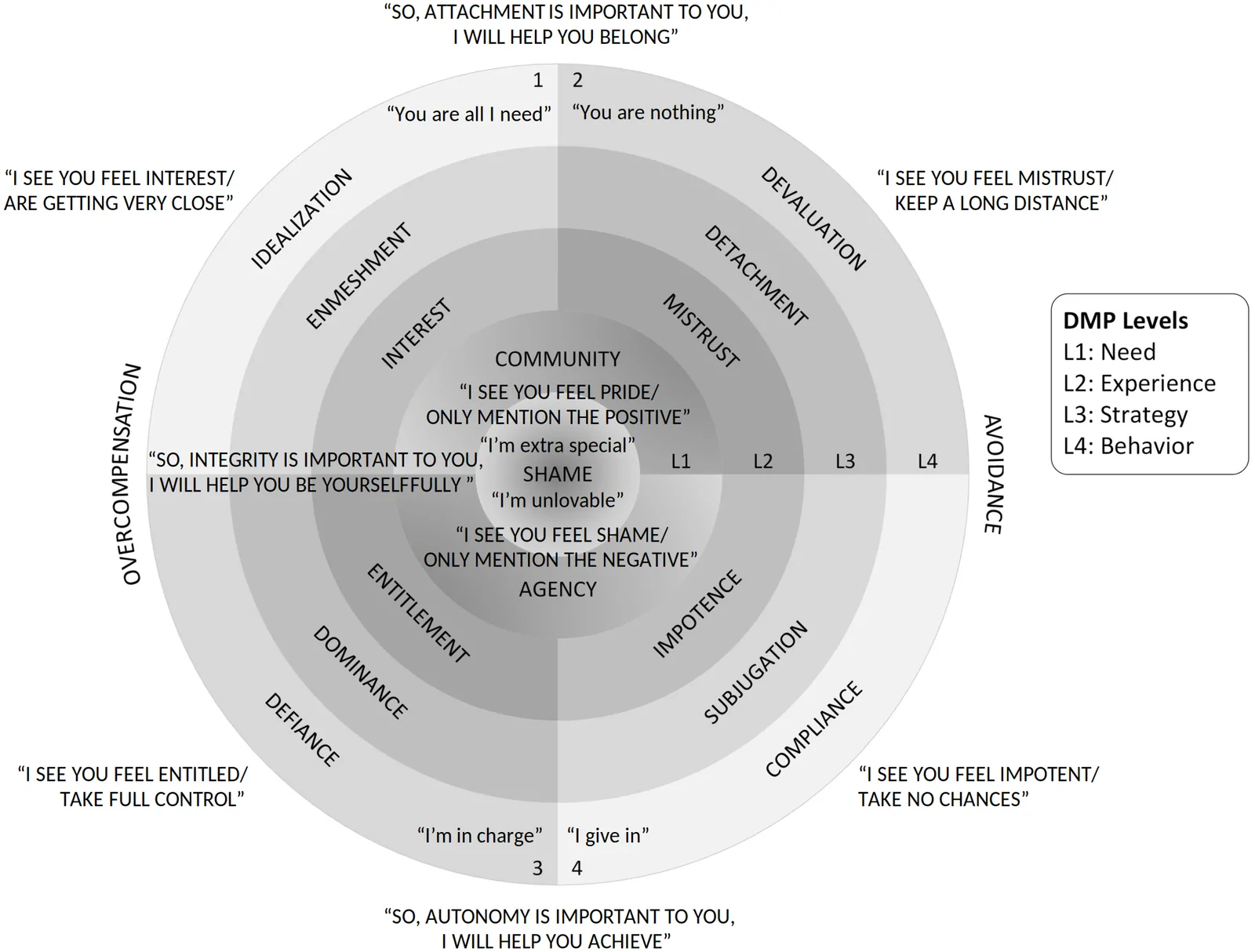
The Dynamic Model of Psychopathy (DMP) (49) with implicit communication (lowercase letters) and the therapist’s customized response (uppercase letters) projected onto the model. The DMP (**Figure 1**) understands psychopathy three-dimensionally, organized into four sectors around a vertical needs-axis (community; agency) and a horizontal defense-axis (overcompensation; avoidance). The model specifies a core of shame and four outer levels comprising relational needs (L1), inner experiences (L2), strategies (L3), and behaviors (L4) associated with psychopathy. The community (affiliation) dimension illustrates how psychopathic individuals alternate between occupying (“you are all I need”) and dismissing (“you are nothing”) relationships. Along the agency (power) dimension, the individual will shift between defying (“I’m in charge”) and evading (“I give in”) action. The four sectors illustrate the narcissistic and anxiety-reducing compensation to meet relational needs and to keep shame associated with a lack of community and agency at a distance. The DMP core and self- and affect consciousness dimension is, thus, also dynamic. As regard the fully compensated self-image and affect, the psychopathic individual says it best, “I’m magnificent” (unconscious shame). An awareness of insignificance (conscious shame) represents the opposite extreme. Adapted from Gullhaugen et al., (2021). The theoretical validation of the Dynamic Model of Psychopathy (DMP): Toward a reformulation of the construct, assessment, and treatment of psychopathic traits. (c) 2025 APA, all rights reserved. https://doi.org/10.1037/pap0000336.

## 2 Psychopathy is a key to integrating dimensional PD constructs and psychotherapy

The DMP paper (49) presents the model’s three-dimensional structure, organized into four sectors around a vertical needs-axis and a horizontal defense-axis, a core of shame, and four outer levels comprising relational needs, inner experiences, strategies and behaviors associated with psychopathy (**Figure 1**) and the related assessment and treatment logic. By expanding the DMP from psychopathy to the broader domain of PD (psychopathy as a diagnostic endpoint, integrating dimensional PD constructs, and distinguishing between healthy personality, PD, and other psychiatric disorders), the rudimentary assessment and treatment conceptualizations outlined in the 2021 paper has increasingly become a more sophisticated and robust framework, which is detailed in the subsequent sections.

### 2.1 The Dynamic Model of Psychopathy Assessment and Treatment Framework

The DMP-ATF is tied to the three-dimensional structure of the model. Assessment comes first, as treatment depends on it and should be made-to-measure^12^ for increased precision and better outcome.

#### 2.1.1 DMP assessment

While the PCL-R, in essence, covers half of the content (grandiose, detached, and dominant traits) of the three DMP dimensions, no specific measure captures the dynamic interplay of the affective and interpersonal, conscious and unconscious states that the DMP portrays. The PCL-R provides an atheoretical cut-off for the severe end of psychopathy (PCL-R ≥ 30), as studies establish more serious crimes and affective-interpersonal deficits (callous grandiosity) in individuals scoring above this threshold (15). The DMP makes sense of these findings, as *externalized or unconscious shame, evidenced by an anxiety-reducing compensation or self-enhancement, will hypothetically fail to trigger empathy and to inhibit ruthless behavior* (**Table 1**). Academic record demarcates psychopathy types through anxiety, emotional distress, trauma, and etiology^13^, and the DMP thus explains the apparent lack of anxiety, emotional distress, and trauma in the primary variant or severe dimensional end as a function of emotional awareness. Linking psychopathy types to unconscious and conscious shame is not a radical reinterpretation as the PCL-R includes grandiosity^14^, but highlights a potential difficulty (shame) experienced by psychopathic individuals and provides a foundation for empirical analysis (falsifiable predictions about the presence or absence of self-enhancing versus self-effacing behavior can be made, cf. fn. 14). Shame, conceptualized as a humiliating and acutely self-conscious affect, requires the prior activation of a positive affect and leaves the self-suspended between longing and despair (54), as evidenced by the DMP needs and defense axes (**Figure 1**). The DMP is thus rigged for multisurface interpersonal assessment (55), which can be useful in measuring personality functioning (56)^15^ and for precision in prognostic formulation and intervention. **Table 2** presents the hypothesized relationship between the type or degree of psychopathy (51, 52) and the integration (reflection on or awareness of intrinsic experiences or states, value and uniqueness) (32, 33, 57–59)^16^, communication (implicit; explicit) (60), and assessment (indirect; direct) (49) of shame (DMP core).

**Table 2:** The hypothesized relationship between the type or degree of psychopathy and the integration, communication, and assessment of shame.

Psychopathy^1^	Integration^2^	Communication^3^	Assessment^4^
Primary	Unconscious	Implicit	Indirect
Secondary	Conscious	Explicit	Direct

^1^ Primary and secondary psychopathy is defined through anxiety, emotional distress, trauma, and etiology (51, 52), including shame and its associated anxiety. ^2^ Based on the sparse literature available, shame is hypothesized to be externalized or unconscious in primary psychopathy (32, 33, 57–59) ^3^ While secondary psychopathy is associated with explicit communication of emotional distress (51, 52), this can be implicitly communicated in primary psychopathy, i.e., through physical characteristics and a lack of coherence (60). ^4^ While secondary psychopathy is associated with direct assessment of emotional distress (51, 52), this can be indirectly assessed by instruments measuring grandiosity (49).

Rather than specific behaviors (e.g., criminal and promiscuous, as included in the PCL-R), broader interactional tendencies (idealization; devaluation; defiance; compliance) are included in the DMP (**Figure 1**). This makes the model pertinent across populations, including different age groups (a delay in achieving developmental psychological milestones was hypothesized as a prerequisite, which aligns closely with the requirement that diagnostic impairments in self and interpersonal functioning must exhibit a clear deviation from age-appropriate norms and manifest as a stable, long-term pattern as opposed to transitory developmental stages) (4), albeit more difficult to score. To improve the interrater reliability, the behavioral level was converted into more concrete verbal and nonverbal behavioral targets (occupies; dismisses; defies; evades) during an institutional implementation course (not intended for publication) and a course of individual psychotherapy currently spanning 3 years (53, 61)^17^, as were the core (self-enhancement; self-effacement). All tendencies should be measured over time, places, individuals, and circumstances (ecological momentary assessment) (63), to make assessment truly dynamic. Observations (verbal; non-verbal) of personality pathology and behaviors in accordance with treatment goals can be summarized qualitatively and quantitatively. In **Figure 2**, ecological momentary assessment of behaviors that are characteristically present in therapy sessions with psychopathic individuals are included for illustrative purposes, while self-report and staff ratings of the same tendencies are alternative approaches to capturing transient or fluctuating states. Crucially, the task is not to extinguish all tendencies but making extreme, rigid, and volatile forms of such actions (e.g. to impose or force oneself on others; DMP sector 1) more adaptive over time (e.g., greeting someone, also sector 1). Indeed, the affiliation and control dimensions of Leary’s IC, which the DMP community and agency dimensions reflect, places PD symptoms in a universal context. It is the combination and the intensity (or distance from origo) of an individual’s placement on the IC that defines the behavior as maladaptive or not. As such, *the DMP-ATF is hypothesized to personalize treatment regardless of PD configuration and the distance to the treatment* goals (**Table 1**).

**Figure 2:**
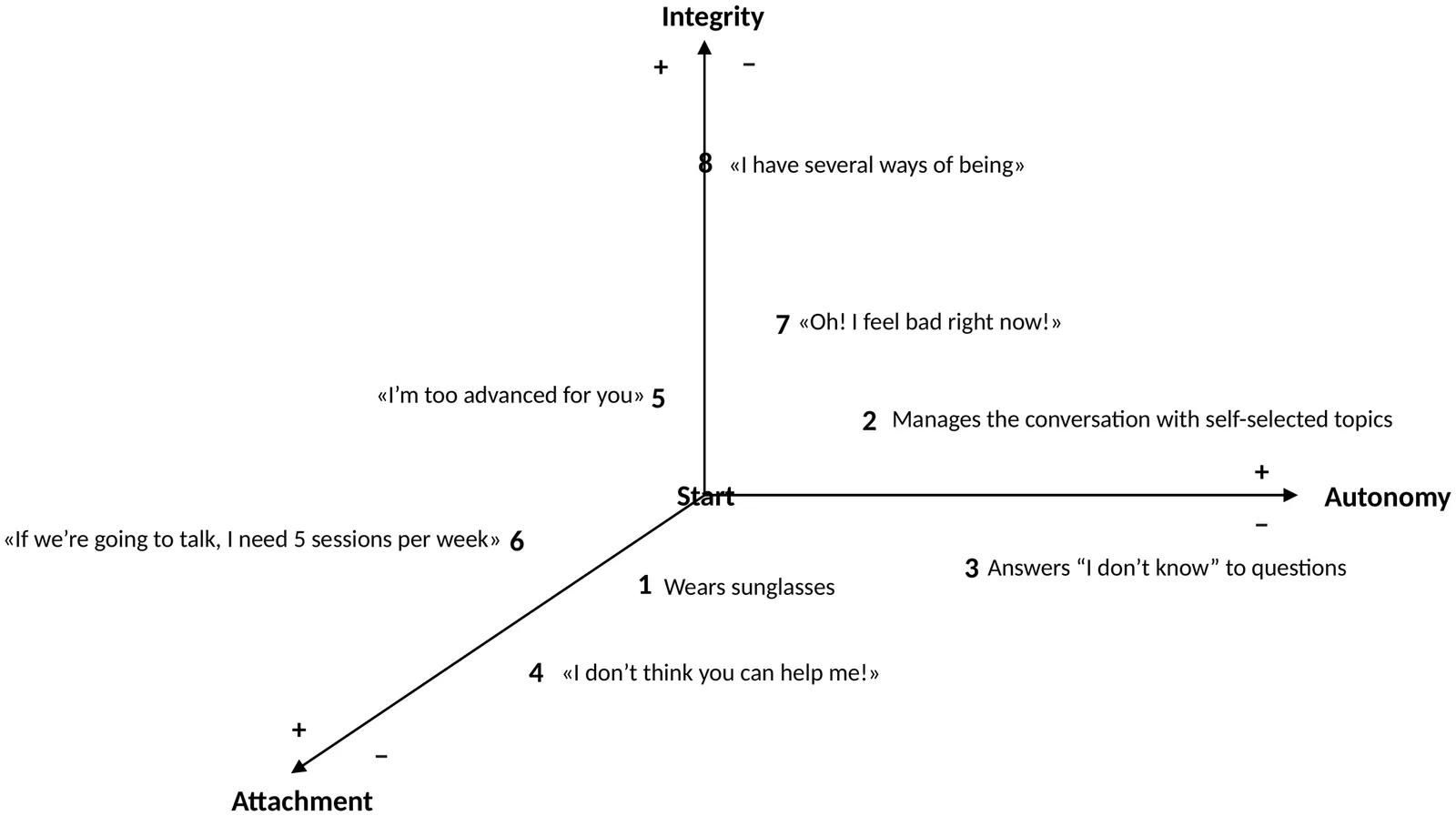
Ecologically momentary assessment of behaviors that are characteristically present in therapy sessions with psychopathic individuals. **Figure 2** illustrates how verbal and non-verbal behavior that are characteristically present in therapy sessions with psychopathic individuals can be displayed according to the DMP dimensions. In this figure, behavior is registered on the appropriate dimension at the right time (from the center outwards) for an overview of the conversation. The plus and minus signs respectively indicate overcompensation and avoidance behaviors.

If substantiated by empirical evidence, redefining and integrating personality with its disorders could lead to a streamlining of the diagnostic PD nomenclature that might benefit not only therapeutic but also medico-legal settings, as clinicians will require comprehensive training to ensure consistency when adopting the ICD-11 (4). Expert testimonies and criminal responsibility assessments could be predicated on a more comprehensive fundament that allows for longitudinal tracking of symptoms and functioning. Inpatient forensic psychiatric assessment facilitates a comprehensive evaluation of the individual’s capacity for regulation within personal, relational, and occupational spheres, which was hypothesized to follow a different pattern in PD (detailed in section 1.2) than in psychosis. The manifestations of the verbal and nonverbal behaviors that can be measured within the DMP-ATF will, however, differ across populations (gender- and age-specific adaptations of the framework are nonetheless not required, as earlier hypothesized). As an example, the impression management (precursor to the glibness/superficial charm PCL-R item) of the checklist’s youth version (64) and the negative self-image of females with psychopathy (65) are subsumed under the DMP-ATF.

#### 2.1.2 DMP treatment

While several evidence-based methods have long existed for PD in general (e.g., Schema Therapy, ST) (66) and for borderline PD in particular (e.g., Dialectical Behavior Therapy, DBT) (67), experts have more recently started to adapt such approaches to the forensic population from which most psychopathy studies have drawn their samples. Examples are ST for violent PD offenders (68), Compassion-Focused Therapy (CFT) for detained youth with psychopathic traits (59), and Mentalization-Based Therapy (MBT) for offending adult males with antisocial PD (69). Psychotherapy, thus, is anchored in therapeutic models targeting parts of the disorder (32) (DBT: Managing emotions; ST: Pervasive, negative themes about oneself and the world; CFT: Shame/self-criticism; MBT: Ability to mentalize)*^18^* and PD categories, instead of fully leveraging the scope of the dimensional framework (e.g. identity, self-direction, empathy, intimacy; cf. the AMPD level of impairment of self-interpersonal functioning).

The DMP-ATF seeks to encompass^19^ the full range of PD and organize the central focuses of existing methods that briefly, and from a meta-perspective, target individual frailty in understanding and tolerating affect, oneself, and others, while, as Galietta and Rosenfeld (70) called for, increasing the effort on that which is distinctly psychopathic; the profound lack of self- and affect consciousness. Progress, or lack thereof, can be monitored within the framework, an interactive and continuous system as the disorder itself is dynamic and affected by external influences (73) such as treatment. Due to the thoroughness in assessment, a continuous process throughout the course of treatment is recommended, attempts at deception are hypothetically more easily detected. This makes the DMP-ATF potentially valuable in risk management as well, as risk is hypothetically linked to the degree of self- and affect consciousness in the individual^20^. A *low level of emotional awareness is hypothesized to equal high risk of the goal-directed behavior* (using others to satisfy needs) *and the instrumental* (not preceded by an affective reaction) *violence that is characteristic of psychopathy* (74) (**Table 1**). **Figure 3** illustrates the DMP-ATF Risk management ladder, which operationalizes a structured approach to mitigating violent recidivism risk among individuals with severe personality deficits.

**Figure 3:**
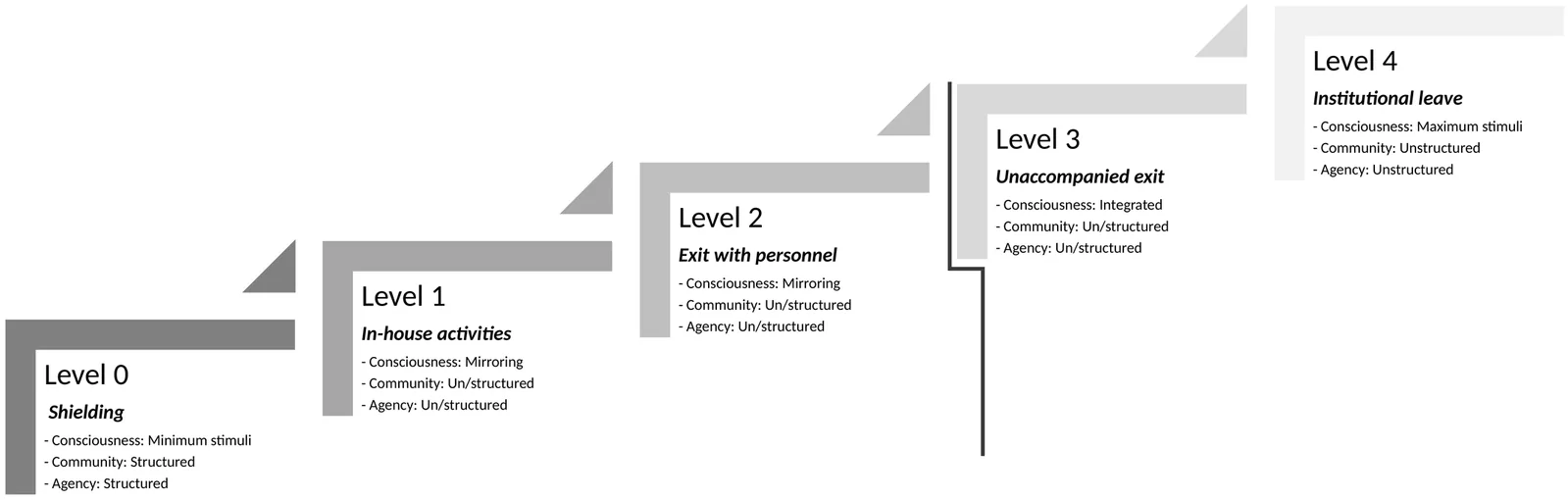
The DMP-ATF Risk management ladder. **Figure 3** presents the DMP-ATF Risk management ladder, where risk is linked to the level of affect consciousness in the individual. The demarcation line between level two and three illustrates that risk is hypothetically lower with integrated affects. The figure further illustrates how an individual’s community and agency can be structured accordingly, and that empathic mirroring is a means to move the individual up the ladder, toward secure belonging and genuine agency.

By measuring individual differences in self- and affect consciousness (self-enhancement-self-effacement), community (occupies-dismisses), and agency (defies-evades), treatment can be personalized and aimed toward the developmental psychological milestones of personal integrity, secure attachment, and genuine autonomy, which are the DMP treatment goals. These are easily assessable, universal concepts, fairly detached from therapeutic models, with the potential of easing collaboration (3). Integrity concerns an individual’s capacity to relate to one’s true self and emotions (75, 76)^21^, implying that psychopathy can be associated with a discomfort in person. Attachment refers to an individual’s capacity to relate to others (secure responsive relations) (77), indicating a discomfort in relationships. Finally, autonomy (healthy independence) (77) affects the capacity to develop skills and competence (representing how individuals react to external pressure), denoting a discomfort in performances. In sum, a *pervasive discomfort* that is constantly compensated for by the individual (**Figure 1**; overcompensation vs. avoidance), potentially explaining the notion that individuals with psychopathy do not suffer (14). Treatment can ignite a natural but delayed process to acquire these relational capacities that most people take for granted, but which requires meticulous and fruitful interaction over time to truly accomplish (33, 54). On that note, *treatment within the DMP-ATF is a potential high precision tool hypothesized to significantly attenuate treatment duration and mitigate the risk of relapse* (**Table 1**). Importantly, treatment within this framework does not require adherence to some manual or patient course (which should not overshadow the considerable demands imposed by the method)^22^, but rather constant evaluation of behavior regarding to its placement on the DMP dimensions and the need and discomfort that is implicitly communicated (unintegrated). In this, the prosocial orientation behind the ruthless behavior becomes evident, and treatment starts instantly as therapists respond therapeutically from the first encounter. This comes with a potential regulatory effect and might work like a brake even if it is not, which is the point. Emotions and needs are not frustrated, and individuals are not subjected to public disgrace.

A system for converting externalizing behaviors into self-conscious emotions and needs by utilizing a mirroring technique (**Figure 1**) can bring out the vulnerability and pain in those characterized by the suffering they impose on others^23^ and make treatment a tangible but counterintuitive and demanding process^24^. Confrontation, along with being overwhelmed (78) are seemingly instinctual responses^25^. Confrontation, however, tends to escalate (more externalization) a situation, at least in laboratory studies (80), while a focus on externalized emotions and needs has a potentially de-escalating^26^ and integrative effect as the individual is met with understanding. In the face of transgressive behaviors, however, it can become difficult to think clearly (“cognitive shock”) (36). The DMP serves as a roadmap in these situations, as the drives behind the behavior or “mask” can be put into words.

In treatment, needs and compensation are initially (and continuously) assessed, and the main tendency (e.g. dominance, DMP sector 3) is met by attuning to and reflecting the underlying emotion (“I see you feel entitled”, cf. **Figure 1**), which can be integrated on various levels (e.g. from action tendencies and undifferentiated affect, here; to speak continuously, to emotional awareness of a single emotion, here; entitlement) (48), which potentially ceases the behavior (regulation, not setting boundaries), which again makes room for other strategies and behaviors (sound choices) that might increase the likelihood for needs satisfaction (here; agency). This is a circular process and a continuous cycle where outputs become inputs. This is also an organic process that mimics natural relations and development; no one is born a finished product (opposite of biological determinism). Changes in states over time will hypothetically result in changes in traits, which may also apply in risk assessment (risk might be lowered one integrated affect at a time). This sounds easy in theory but is very challenging in real life, with each case demanding its own unique parameters.

In difficult cases, starting at the strategy level (“I see you take full control”) and following up with the need (“so, autonomy is important to you”) may be a useful advice, as needs are motivational and as strategies might be more consciously available and less anxiety-provoking than emotions. In this process, individual therapists and staff are easily challenged beyond their capacity, which is observed to sometimes result in excessive talking off topic (DMP sector 3), highlighting achievements even when unsuccessful (core; self-enhancement), division between colleagues (sector 2), arguing that the method is ineffective (sector 4), and feeling like a failure (core; self-effacement). In this way, *professionals* might temporarily lose their integrity, which explains why psychopathy is hard to treat. Fighting fire with fire in staff is unproductive; the mirroring techniques might work here as well.

Regulation is essential (71), not constraining (which at times is nevertheless also needed). The “not-knowing” stance (asking questions) of MBT (82) comes naturally to the staff and many therapists but is probably not effective when the conscious component of an emotion is out of reach (cf. the levels of emotional awareness by Lane & Smith, level 2, for action tendencies) (48). The capacity of the therapist to be with and expand the patient’s emotions^28^, also included in MBT (and CFT), might help. Through marked mirroring^29^ of emotions and precise interpretation of needs in appropriate doses over time, the individual discovers him- or herself^30^ through the eyes of the other. This is beneficial due to the antidialogical nature of shame. That which is shameful is difficult to put into words and might pull the person into hiding. This applies not only but particularly to psychopathic individuals who hide their “true face” (tentatively, DMP needs, experiences, and strategies) behind a mask (14). Hypothetically, all PDs could be different faces of shame^31^ (83–85), as community and agency to a greater or lesser degree are compromised and can be increased (86) in all variants. Hence, the DMP-ATF is not only potentially relevant to the most pervasive PD level that psychopathy might be, but for more limited types as well, for which parts of the framework can be utilized.

In sum, in the second part of this article it is sought to establish how psychopathy can be a key to integrating dimensional PD constructs and psychotherapy by elevating shame to a primary objective (the analysis extends its diagnostic and clinical utility beyond psychopathic manifestations), and by grounding treatment in universal concepts outside PD categories and therapeutic models. These can be vital components in personological maturation (43), hypothesized (**Table 1**) as individual balance and thus variance on three dimensions, whose healthy solution might bring equanimity to disrupted personalities. Finally, all arguments presented in this article require empirical validation.

## 3 Discussion: toward a reformulation of personality and its disruption

In this article it is argued that psychopathy is a key to integrating dimensional PD constructs and psychotherapy. Novel arguments and interpretations are presented throughout the paper, while the research questions and hypotheses are organized in **Table 1** by the order of appearance in the article.

In section 1.1 it was argued that experts fail to recognize conceptual relevance in the extant literature, suggesting that a self- and affect consciousness dimension is required besides the IC to explain all PD variants. This led to the hypothesis that psychopathy represents the most disruptive or self-protective manifestation of the dimensions, evidenced by grandiose, detached, and dominant tendencies and less frequent shifts on the IC relative to borderline PD, as shame operates unconsciously. In section 1.2 it was argued that the proposed self- and affect consciousness dimension is compatible with the DSM-5 AMPD and the ICD-11 PD sections positing identity problems and self-functioning impairments; that deficits in self- and affect consciousness can explain the lack of predominantly affective empathy that is a hallmark of psychopathy; that the empathy functioning impairment of the AMPD and the ICD-11 focusing on perspective taking might be redundant, and; that the remaining DSM-5 and ICD-11 functioning impairments can be organized under self- and affect consciousness or as belonging to Leary’s IC dimensions. On this basis, it was interpreted that the self- and affect consciousness dimension could serve to simplify the diagnostic nomenclature, culminating in the hypothesis that the AMPD and the ICD-11 functioning impairments can be organized according to the self- and affect consciousness dimension and Leary’s dimensions of affiliation and power.

In section 1.2 it was further argued that that the dimensions that are required to predict PD can be utilized to clarify personality, and that a healthy personality would involve balance not rigidity in or shifts between extreme states of self- and affect consciousness, affiliation, and power. This led to the hypothesis that individual balance and thus variance on the three dimensions can serve as an index or metric of personological maturation or personality. It was then argued that identifying a diagnostic endpoint does not intervene with the moving away from categorical descriptions to the idea that the degree of PD is more central than the type, but may define the boundaries of movement. This led to the hypothesis that PD is uniquely distinguished from other mental disorders by a structural imbalance within integrity, attachment, and autonomy, manifested as rigidity and shifts between extreme states, whereas other conditions maintain a degree of individual balance. The argument that the DMP might counter the lack of an integrated understanding that has been identified as a major cause of the treatment trouble associated with the disorder, by explicating a dynamic interplay of states hypothesized to transcend populations, age, and severity levels as parts of universal developmental psychological processes, concluded the section.

In section 2.1.1 it was argued that the DMP makes sense of the PCL-R’s atheoretical cut-off for severe psychopathy, by explicating how unconscious shame, evidenced by anxiety-reducing compensation or self-enhancement will fail to trigger empathy and to inhibit ruthless behavior, which was hypothesized. It was further argued that by placing PD symptoms in an universal context, the DMP-ATF can be hypothesized to personalize treatment regardless of PD configurations and the distance to the treatment goals. In section 2.1.2 it was argued that the thoroughness in assessment makes attempts at deception more easily detectable. As such, the DMP-ATF is potentially valuable in risk management as well, as risk can be linked to the degree of self- and affect consciousness in the individual, leading to the hypothesis that diminished emotional awareness elevates the risk for the goal directed behavior and the instrumental violence characteristically observed in psychopathic individuals. Finally it was argued that current psychotherapies target parts of the disorder instead of fully leveraging the scope of the dimensional framework, leading to the hypothesis that the thorough and continued assessment associated with developmental psychological milestones and the focus on states and emotional awareness can reduce treatment duration and the risk of relapse, which would make the DMP-ATF a high precision tool. In sum, the proposed hypotheses in this article offer three potential main contributions; a unifying framework in diagnostic classification of PD (awaiting construct validity), a delimitation of how PDs are different from personality and other mental disorders (awaiting discriminant validity), and a treatment framework with superior precision in facilitating the shift from PD to adaptive personality structures (awaiting incremental validity), which together with more subordinate research questions and hypotheses are inserted in **Table 1**.

Indeed, the DMP-ATF is currently a theoretical framework that requires empirical validation. The DMP-ATF is, however and as demonstrated, in line with current developments in the PD field. Greater clarity is, nevertheless, still needed regarding the shift from descriptive, symptom-based criteria toward dimensional measures (1), and the latent constructs of the DMP-ATF can be a step in this direction. Further, dimensionality is claimed to have brought the diagnostic language closer to lived experience and to the processes targeted in therapy (87). The DMP-ATF can be argued to continue to operate within this realm, hypothetically with higher precision. Importantly, clinicians should adapt their practices to the fact that individuals with PD vary in the degree to which they are capable of participating in conventional treatment. The etymological root of shame is to hide oneself, which makes sense in this context. Novel information about what PD really is (tentatively; lacks in integrity, attachment, and autonomy) and is *not* (evil, immorality, non-treatable) might increase help-seeking behavior and ensure that interventions yield substantive benefits. Access to high-quality self-help materials may facilitate entry into formal treatment and prove sufficient for some individuals. Altogether, parts of the massive financial and human cost on society due to PD can possibly be saved (88)^32^. On that note, psychopathy conceptually rooted in callous infliction of suffering has posed a formidable clinical challenge, but reframing psychopathy through a lens of pain and vulnerability (89, 90) might be the key to integrating dimensional PD constructs and psychotherapy.

## Data Availability

The original contributions presented in the study are included in the article/supplementary material. Further inquiries can be directed to the corresponding author.

## Footnotes

1 The PCL-R items promiscuous sexual behavior and many short-term marital relationships do not load on any factor (15), potentially indicating that the concept does not fully account for the disorder.

2 While initially compelling, basing the CAPP on a lexical approach overlooks the reality that most individuals remain constrained by their prevailing cognitive paradigms, suggesting that core conceptual aspects are yet to be elucidated.

3 Psychopathic antisocial subjects “present a malevolent, destructive internalized object world characterized by intense and violent intrapsychic conflict surrounding attachment and separation” (p. 46) (31).

4 Prosser et al. (32) provide a formal, computational model of this process, suggesting that “lacks remorse” is not a lack of emotion, but a result of abnormal inferences aimed at maintaining high self-worth and avoiding discomfort. Meloy (33) stressed how “the reptilian state is not a characterological trait. It is a functional psychobiological state that momentarily may exists with its object relational and instinctual correlates” (p. 75), cf. the current focus on functional definitions (34).

5 AMPD identity problems: Experience of a unique self with clear boundaries from others, stability of self-esteem and accuracy of self-appraisal, the capacity to experience and regulate a full range of emotions; ICD-11 self-functioning impairment: Identity, self-worth, self-direction, and self-perception and their manifestations and expressions.

6 Per Nathanson, “shame affect causes a “cognitive shock”, a momentary inability to think clearly” (36) (p. xxvi).

7 Hutsebaut et al. (1) state that “the endpoint of the mechanisms of change in psychotherapies is consistent with good personality functioning, as balanced and nuanced emotional experience of self-in-interaction with others, with clear but semiopen boundaries of identity and a capacity to narrate one’s life story in a self-reflective and wise manner” (p. 5).

8 “More abrupt or extreme shifts in a personality dimension (at the level of *state* changes), such as dominance, can be observed in interactions with patients who have more impaired global personality functioning” (p. 6) (1) . 

9 Personality should merge with developmental psychopathology to a larger degree (41).

10 This definition can be related to the original usage of the personality construct, a mask worn by actors in ancient Roman theatres, where, conceivably, all kinds of characters were portrayed.

11 Zavlis (42) challenged Hopwood et al.’s (43) view of personality functioning as a one-dimension axis involving psychological maturity, as specific domains in psychological development cannot be coherently reduced to one process (i.e., basic trust and autonomy), which is in accordance with the three-dimensional framework set forth in this article.

12 The study of complex interaction patterns or a type of profiling “would allow clinicians to improve prognoses and personalize predictions about treatment outcomes” (1), p. 4.

13 Karpman (51) separated psychopathy into idiopathic (congenital; profound deficits in emotional depth, empathy, and conscience, a low baseline of anxiety, calculating, charming, and manipulative, detachment from emotional states) and symptomatic (acquired; a higher degree of anxiety, internal conflict, depression, or emotional reactivity; the antisocial behavior serves as a symptomatic manifestation of deeper psychological distress or vulnerability) types, more commonly recognized as primary versus secondary variants of the disorder and more recently situated on the same dimension (52).

14 Grandiosity can be defined as an unrealistic, inflated sense of superiority, self-importance, and uniqueness that is not based on actual capability, and as such, hypothesized manifestations of compensated or unconscious shame. Persistent self-enhancement or grandiosity across contexts without independent evidence of shame or other negative self-conscious affect, including when the self-concept is challenged, would count against the hypothesis that shame underlies primary or severe psychopathy (the important falsifiable component is whether the proposed self-enhancement can occur in the absence of the underlying shame process that the model posits). Grandiosity is, however, hypothesized to be the *main tendency* on the self- and affect conscious dimension in severe psychopathy, while negative affect may intermittently occur due to the dynamic nature of self-states (33) and hypothetically as a result of treatment (53).

15 Gilbert and Hopwood (56) identified cross-surface discrepancy in that individuals who tend to be too cold (i.e., the *behaviors* they perform excessively, cf. the DMP behavior level, **Figure 1**) have higher levels of personality functioning to the degree they *value* and engage in non-cold behaviors (i.e., desired interpersonal outcomes, cf. the DMP needs level).

16 Meloy (33) summarized the historical antecedents of intrapsychic dynamics in psychopathy and noted, within a broader context, that “the shameful, worthless selfobject is expelled and projected onto the actual victim; and the psychopathy is subsequently exhilarated during this perceived victory as the fantasied grandiose self is cleansed and glorifies” (p. 10).

17 Having already presented at international conferences, this therapy is currently being prepared for publication as a peer-reviewed article (62), and an independent evaluation is underway within the framework of a master’s thesis.

18 Exaggerated for effect.

19 I.e., there is currently no evidence for the broader treatability of psychopathy from evidence for the DMP-ATF specifically. The treatment literature is encouraging but mixed (68–72).

20 Prosser et al. (32) model remorselessness partly as a defense against shame and worthlessness, while Normann-Eide et al. (58) discusses evidence linking psychopathy to low affect consciousness specifically for shame. Jantzi and Moulin (4) emphasize that the focus on severity (reflecting the degree of dysfunction in self and interpersonal functioning and the associated risk, e.g., harm to others) is particularly relevant in forensic settings, as it is a major predictor of outcome. The DMP-ATF hypothetically further specifies the (degree of) dysfunction in self and interpersonal functioning.

21 Experiences shape a coherent sense of self in contrast to fragmented states (76).

22 While formal publication authorization remains pending, this article nevertheless synthesizes the overarching insights gained from a three-year trial of the DMP-ATF within the Norwegian child welfare sector.

23 According to Meloy (33) p. 71, “The reptilian, predatory eyes are, in a sense, the antithesis of the affectionate mirroring of the infant in the eyes of the mother. The nascent self is reflected as an object of prey, rather than an object of love. The fixated stare of the psychopath is a prelude to instinctual gratification rather than empathic caring. The interaction is socially defined by parameters of power rather than attachment”.

24 Children “wear” their emotions, which are easily mirrored for developmental purposes. The emotions of psychopathic individuals are difficult to mirror as they are hypothesized to “hide behind a mask”. Focusing on the three DMP dimensions might prevent the emergence of empathy deficit or mentalization failure *in therapists.*

25 Luo et al. (79) identified distinct interpersonal patterns associated with alliance ruptures. In this single case, the patient’s warmth (cf. DMP sector 1) was positively related with withdrawal ruptures concurrently in the session.

26 In crisis and hostage negotiations, mirroring is considered to come closest to a Jedi mind trick (81).

28 For those cooperating, the emotions associated with any given tendency (e.g. entitlement, DMP sector 3) can be mirrored, which gives a here-and-now focus. This allows for an emotional bridge to be drawn between present-day experiences (entitlement) to past emotions (here; impotence, DMP sector 4), which again can be linked to the future (the importance of sustainable, genuine autonomy/treatment goal). Shame and anxiety is also a part of this mix. As such, the DMP-ATF is arguably and hypothetically a precision tool providing essential guidance for clinicians in a new area.

29 When the therapist mirrors the patient in a *supportive* and *calming* way, not mimicking nor mocking. It is important to not only repeat the same sentence, e.g., “I see that you do not trust people” and to adapt the wording to the recipient, as inflexibility on the part of the therapist in this area might prevent therapy from getting started.

30 Emotions are essential parts of the self. Therefore, emotional distance signifies self-distance; the individual cannot be and is not perceived as authentic or genuine, i.e., as an individual with personal integrity or a healthy personality.

31 In their study, Asgarizadeh and Ghanbari (83) focused on cluster B, while Dickinson and Pincus (84) reported shame in vulnerable (not grandiose) narcissistic individuals who were represented by high ratings of avoidant PD.

32 Antisocial behavior and violence risk contributed strongly to those costs and the sample was male and forensic.

## References

[1] Hutsebaut J Sharp C Kramer U . Dimensional construct of personality disorder: Implications for psychotherapy integration. J Psychother Integration. (2026) 36:1–9. doi: 10.1037/int000039227371692

[2] Monaghan C Bizumic B . Dimensional models of personality disorders: Challenges and opportunities. Front Psychiatry. (2023) 14:1098452. doi: 10.3389/fpsyt.2023.1098452 36960458 PMC10028270

[3] Duggan C . The contribution of interpersonal theory to personality disorder's diagnosis and treatment. BJPsych Adv. (2026) 32:257–65. doi: 10.1192/bja.2025.10187

[4] Jantzi C Moulin V . The new ICD-11 diagnosis of personality disorder in forensic psychiatry. Front Psychiatry. (2025) 16:1630512. doi: 10.3389/fpsyt.2025.1630512 40904569 PMC12401950

[5] American Psychiatric Association . Diagnostic and Statistical Manual of Mental Disorders (5th ed.). (2013). Washington, DC: American Psychaitric Association.

[6] World Health Organization . International Statistical Classification of Diseases and Related Health Problems (11th ed.). (2019). Geneva, Switzerland: World Health Organization.

[7] Zavlis O Moutoussis M Fonagy P Story GW . A generative model of personality disorder as a relational disorder. J Psychopathol Clin Sci. (2025) 135:25–46. doi: 10.31234/osf.io/wh6na_v2 40705631

[8] Kotov R Krueger RF Watson D Achenbach TM Althoff RR Bagby RM . The Hierarchical Taxonomy of Psychopathology (HiTOP): A dimensional alternative to traditional nosologies. J Abnormal Psychol. (2017) 126:454–77. doi: 10.31234/osf.io/zaadn 28333488

[9] Poore HE Waldman ID . Associations among externalizing psychopathology, personality, and behavioral traits: Models of an externalizing spectrum in youth. Pers Disorders: Theory Res Treat. (2025) 16:8–17. doi: 10.1037/per0000686 39818927 PMC12290986

[10] Blackburn R Logan C Renwick SJD Donnelly JP . Higher-order dimensions of personality disorder: Hierarchical structure and relationships with the five-factor model, the interpersonal circle, and psychopathy. J Pers Disord. (2005) 19:597–623. doi: 10.1521/pedi.2005.19.6.597 16553558

[11] Wright AGC Ringwald WR Hopwood CJ Pincus AL . It's time to replace the personality disorders with the interpersonal disorders. Am Psychol. (2022) 77:1085–99. doi: 10.31234/osf.io/7syvf 36595407

[12] Kennedy TD Anello E Sardinas S Woods SP . Recognizing a psychopath: conceptual confusion. In: Working With Psychopathy. Cham, Switzerland: Springer (2021).

[13] Larsen RR . Psychopathy treatment and the stigma of yesterday's research. Kennedy Institute Ethics J. (2019) 29:243–72. doi: 10.1353/ken.2019.0024 31656233

[14] Cleckley HM . The Mask of Sanity: An Attempt to Clarify Some Issues About the So-Called Psychopathic Personality. St. Louis, Missouri: Mosby (1941).

[15] Hare RD . Manual for the Revised Psychopathy Check List (2nd Ed.). Toronto, ON, Canada: Multi-Health Systems (2003).

[16] Patrick CJ Fowles DC Krueger RF . Triarchic conceptualization of psychopathy: Developmental origins of disinhibition, boldness, and meanness. Dev Psychopathol. (2009) 21:913–38. doi: 10.1017/s0954579409000492 19583890

[17] Cooke DJ Hart SD Loagan C Michie C . Explicating the construct of psychopathy: Development and validation of a conceptual model, the Comprehensive Assessment of Psychopathic Personality (CAPP). Int J Forensic Ment Health. (2012) 11:242–52. doi: 10.1080/14999013.2012.74675937339054

[18] Polaschek D Skeem JL . Treatment of adults and juveniles with psychopathy. In: Patrick CJ , editor. Handbook of Psychopathy. New York, New York: The Guilford Press (2018).

[19] Sheehan L Nieweglowski K Corrigan P . The stigma of personality disorders. Curr Psychiatr Rep. (2016) 18:1–7. doi: 10.1007/s11920-015-0654-1 26780206

[20] Blackburn R Coid J . Psychopathy and the dimensions of personality disorder in violent offenders. Pers Individ Dif. (1998) 25:129–45. doi: 10.1016/s0191-8869(98)00027-0

[21] Howard RC Khalifa N Duggan C . Antisocial personality disorder comorbid with borderline pathology and psychopathy is associated with severe violence in a forensic sample. J Forensic Psychiatry Psychol. (2014) 25:658–72. doi: 10.1080/14789949.2014.94379737339054

[22] Bergstrøm H Farrington DP Coid JW . Relationships between psychopathy and DSM personality disorders in men and women. Int J Offender Ther Comp Criminology. (2022) 68:278–95. doi: 10.1177/0306624X221094993 35574634

[23] Hildebrand M de Ruiter C . PCL-R psychopathy and its relation to DSM-IV axis I and II disorders in a sample of male forensic psychiatric patients in The Netherlands. Int J Law Psychiatry. (2004) 27:233–48. doi: 10.1016/j.ijlp.2004.03.005 15177992

[24] Khalifa N Howard RC . Is PCL-R psychopathy associated with either type or severity of personality disorder? J Forensic Psychiatry Psychol. (2015) 26:862–77. doi: 10.1080/14789949.2015.107257537339054

[25] Wiggins JS Pincus AL . Conceptions of personality disorders and dimensions of personality. psychol Assess. (1989) 1:305–16. doi: 10.1037/1040-3590.1.4.30527371692

[26] Leary TF . Interpersonal Diagnosis of Personality. New York, New York: Ronald Press (1957).

[27] Wright AGC Hallquist MN Morse JQ Scott LN Stepp SD Nolf KA . Clarifying interpersonal heterogeneity in borderline personality disorder using latent mixture modeling. J Pers Disord. (2013) 27:125–43. doi: 10.1521/pedi.2013.27.2.125 23514179 PMC3607958

[28] Russel JJ Moskowitz DS Zuroff DC Sookman D Paris J . Stability and variability of affective experience and interpresonal behavior in borderline personality disorder. J Abnormal Psychol. (2007) 116:578–88. doi: 10.1037/0021-843X.116.3.578 17696713

[29] Schimmenti A Passanisi A Pace U Manzella S Di Carlo G Caretti V . The relationship between attachment and psychopathy: A study with a sample of violent offenders. Curr Psychol. (2014) 33:256–70. doi: 10.1007/s12144-014-9211-z30311153

[30] Nørbech PC Crittenden PM Hartmann E . Self-protective strategies, violence and psychopathy: Theory and a case study. J Pers Assess. (2013) 95:571–84. doi: 10.1080/00223891.2013.823441 23980823

[31] Gacono CB Meloy JR Berg JL . Object relations, defensive operations, and affective states in narcissistic, borderline, and antisocial personality disorder. J Pers Assess. (1992) 59:32–49. doi: 10.1207/s15327752jpa5901_4 1512679

[32] Prosser A Friston KJ Bakker N Parr T . A Bayesian account of psychopathy: A model of lacks remorse and self-aggrandizing. Comput Psychiatry. (2018) 2:92–140. doi: 10.1162/cpsy_a_00016 30381799 PMC6184370

[33] Meloy JR . The Psychopathic Mind: Origins, Dynamics, and Treatment. Northvale, New Jersey: Jason Aronson (1998).

[34] Wright AGC Hopwood CJ . Integrating and distinguishing personality and psychopathology. J Pers. (2022) 90:5–19. doi: 10.1111/jopy.12671 34480760

[35] Goodhew SC Edwards M . On the relationship between psychopathic traits and cognitive empathy, affective empathy, and empathic concern. Pers Individ Dif. (2025) 247:1–4. doi: 10.1016/j.paid.2025.11344542574925

[36] Nathanson DL . Prologue. In: Tomkins SS , editor. Affect, Imagery, Consciousness: The Complete Edition (Vols 3 and 4). New York, New York: Springer (2008).

[37] Sharp C Clark LA Balzen KM Widiger T Stepp S Zimmerman M . The validity, reliability and clinical utility of the Alternative DSM-5 Model for Personality Disorders (AMPD) according to DSM-5 revision criteria. World Psychiatry. (2025) 24:319–40. doi: 10.1002/wps.21339 40948060 PMC12434376

[38] Trull TJ Widiger TA . Dimensional models of personality: The five-factor model and the DSM-5. Dialogues Clin Neurosci. (2013) 15:135–46. doi: 10.31887/dcns.2013.15.2/ttrull 24174888 PMC3811085

[39] Weekers LC Hutsebaut J Rovers JMC Kamphuis JH . Head-to-head comparison of the alternative model for personality disorders and Section II personality disorder model in terms of predicting patient outcomes 1 year later. Pers Disorders: Theory Research Treat. (2024) 15:101–9. doi: 10.1037/per0000637 37535548

[40] Nysaeter TE Hummelen B Christensen TB Eikenaes IUM Selvik SG Pedersen G . The incremental utility of criteria A and B of the DSM-5 alternative model for personality disorder for predicting DSM-IV/DSM-5 section II personality disorders. J Pers Assess. (2023) 105:111–20. doi: 10.1080/00223891.2022.2039166 35285763

[41] Tackett JL Katz BA . P is for personality: Commentary on Caspi et al. (2026). J Psychopathol Clin Sci. (2026) 135:526–7. doi: 10.1037/abn0001092 42166289

[42] Zavlis O . Psychoanalytic theories do not support a unidimensional view of personality functioning. Pers Ment Health. (2026) 20. doi: 10.1002/pmh.70095 42583936 PMC13463666

[43] Hopwood CJ Driver CC Morey LC Skodol AE . Personality functioning as generalized correlated changes in personality traits. J Psychopathol Clin Sci. (2025) 135:297–305. doi: 10.31234/osf.io/n63cg_v1 41066267

[44] Kernberg OF . A psychoanalytic classification of character pathology. J Am Psychoanalytic Assoc. (1970) 18:800–22. doi: 10.1177/000306517001800403 5493243

[45] Kernberg OF Michels R . Borderline personality disorder. Am J Psychiatry. (2009) 166:505–8. doi: 10.1176/appi.ajp.2009.09020263 19411373

[46] Kernberg OF . Aggressivity, Narcissism, and Self-Destructiveness in the Psychotherapeutic Relationship: New Developments in the Psychopathology and Psychotherapy of Severe Personality Disorders. New Haven: Yale University Press (2004).

[47] Liu Y Zhou B Wendt LP Edershile EA Yang B Wright AGC . Dynamic processes in narcissism and affect across Chinese and American individuals: A registered report. J Pers Soc Psychol. (2026). doi: 10.1037/pspp0000612 42752112

[48] Lane RD Smith R . Levels of emotional awareness: Theory and measurement of a socio-emotional skill. J Intell. (2021) 9:42. doi: 10.3390/jintelligence9030042 34449662 PMC8395748

[49] Gullhaugen A Heinze P Chernyahovsky Kornev S . The theoretical validation of the Dynamic Model of Psychopathy (DMP): Toward a reformulation of the construct, assessment, and treatment of psychopathic traits. Psychoanalytic Psychol. (2021) 38:290–9. doi: 10.1037/pap000033627371692

[50] Lazarus G Rafaeli E . Modes: Cohesive personality states and their interrelationships as organizing concepts in psychopathology. J Psychopathol Clin Sci. (2023) 132:238–48. doi: 10.1037/abn0000699 37126057

[51] Karpman B . On the need of separating psychopathy into two distinct clinical types: The symptomatic and the idiopathic. J Criminal Psychopathol. (1941) 3:112–37.

[52] Skeem JL Polaschek DLL Patrick CJ Lilienfeld SO . Psychopathic personality: Bridging the gap between scientific evidence and public policy. psychol Sci Public Interest. (2011) 12:95–162. doi: 10.1177/1529100611426706 26167886

[53] Gullhaugen A . (2026). “ The suffering of the psychopath: Data from a 3-year single-case study of normal-population psychopathy within the Dynamic Model of Psychopathy Assessment and Treatment Framework (DMP-ATF)”, in: The Society for the Scientific Study of Psychopathy 11th Biennial Meeting (Dallas, TX), 6–9.

[54] Frank AJ Wilson EA . A Silvan Tomkins Handbook. Foundations for Affect Theory. Minneapolis: University of Minnesota Press (2020).

[55] Daewood S Pincus AL . Multisurface interpersonal assessment in a congnitive-behavioral therapy context. J Pers Assess. (2016) 98:449–60. doi: 10.1080/00223891.2016.1159215 27070943

[56] Gilbert KJ Hopwood CJ . Angular discrepancy and personality functioning across interpersonal circumplex surfaces. Assessment. (2026). doi: 10.31234/osf.io/zgth5_v1 42638209

[57] Heinze P . Psychopathy, unconscious shame and attachment: Considering the psychodynamics of psychopathy. Psychodynamic practice: Individuals groups organizations. (2017) 23:7–32. doi: 10.1080/14753634.2016.1269663

[58] Normann-Eide E Johansen M Normann-Eide T Egeland J Wilberg T . Is low affect consciousness related to the severity of psychopathology? A cross-sectional study of patients with avoidant and borderline personality disorder. Compr Psychiatry. (2013) 54:149–57. doi: 10.1016/j.comppsych.2012.07.003 22998844

[59] Ribeiro da Silva D Rijo D Salekin RT Paulo M Miguel R Gilbert P . Clinical change in psychopathic traits after the PSYCHOPATHY.COMP program: Preliminary findings of a controlled trial with male detained youth. J Exp criminology. (2020) 17:397–421. doi: 10.1007/s11292-020-09418-x30311153

[60] Gullhaugen AS Sakshaug TS . What can we learn about psychopathic offenders by studying their communication? A review of the literature. J psycholinguistic Res. (2019) 48:199–219. doi: 10.1007/s10936-018-9599-y 30151666

[61] Gullhaugen A Aanæs M . (2025). “ Data from a 2-year single-case study of normal population psychopathy within the DMP-ATF”, in: International Society for the Study of Personality Disorder Congress - Unifying personality theory and treatment: Bridging hearts, minds, and methods (Boston, MA), 9–11.

[62] Gullhaugen A . Data from a 3-year single-case study of normal-population psychopathy within the Dynamic Model of Psychopathy Assessment & Treatment Framework (DMP-ATF).

[63] Shiffman S Stone AS Hufford MR . Ecological momentary assessment. Annu Rev Clin Psychol. (2008) 4:1–32. doi: 10.1146/annurev.clinpsy.3.022806.091415 18509902

[64] Forth AE Kosson DS Hare RD . The Hare Psychopathy Checklist: Youth Version (Pcl:Yv). Toronto, Ontario: Multi-Health Systems (2003).

[65] Smith JM Gacono CB Cunliffe TB . Understanding Famale Offenders: Psychopathy, Criminal Behavior, Assessment, and Treatment. San Diego, California: Academic Press (2021).

[66] Young JE Klosko JS Weishaar ME . Schema Therapy: A Practitioner's Guide. New York, New York: Guilford Press (2003).

[67] Linehan MM . Dialectical behavioral therapy: A cognitive behavioral approach to parasuicide. J Pers Disord. (1987) 1:328–33. doi: 10.1521/pedi.1987.1.4.32837804533

[68] Bernstein D Keulen-de Vos M Clercx M De Vogel V Kersten G Lancel M . Schema therapy for violent PD offenders: A randomized clinical trial. Psychol Med. (2023) 53:88–102. doi: 10.1017/S0033291721001161 PMC9874993 34127158

[69] Fonagy P Simes E Yirmiya K Wason J Barrett B Frater A . Mentalization-based treatment for antisocial personality disorder in males convicted of an offence on community probation in England and Wales (Mentalization for Offending Adult Males, MOAM): a multicentre, assessor-blinded, randomised controlled trial. Lancet Psychiatry. (2025) 12:208–19. doi: 10.1016/s2215-0366(24)00445-0 39978982

[70] Galietta M Rosenfeld B . Adapting dialectical behavior therapy (DBT) for the treatment of psychopathy. Int J Forensic Ment Health. (2012) 11:325–35. doi: 10.1080/14999013.2012.74676237339054

[71] Mohajerin B Howard RC . Effects of two treatments on interpersonal, affective, and lifestyle features of psychopathy and emotion dysregulation. Pers Ment Health. (2024) 18:43–59. doi: 10.1002/pmh.1593 37799055

[72] McWilliams N Shedler J . Personality styles and disorders. P axis. In: Lingiardi V McWilliams N , editors. Psychodynamic Diagnostic Manual, Third Edition. New York, New York: Guilford (2026). p. 651–708.

[73] Hopwood CJ Kaurin A Pincus AL . Revitalizing personality disorder research. J Psychopathol Clin Sci. (2026) 135:1–8. doi: 10.1037/abn0001064 41411044

[74] Woodworth M Porter S . In cold blood: Characteristics of criminal homicides as a function of psychopathy. J Abnormal Psychol. (2002) 111:436–45. doi: 10.1037/0021-843x.111.3.436 12150419

[75] Damasio AR . The Feeling of What Happens: Body, Emotion, and the Making of Consciousness. London: Heinemann (1999).

[76] Sroufe LA ed. Relationships, Self, and Individual Adaption. New York: Basic Books (1989).

[77] Bowlby J . Attachment and Loss: Vol. 2. Separation: Anxiety and Anger. New York: Basic Books (1973).

[78] Gazzillo F Lingiardi V Del Corno F Genova F Bornstein RF Gordon RM . Clinicians' emotional responses and PDM P axis personality disorders: A clinically relevant empirical investigation. Psychotherapy. (2015) 52:238–46. doi: 10.1037/a0038799 25868053

[79] Luo X Hopwood CJ Good EW Turchan JE Thomas KM Levendosky AA . Using interpersonal dimensions of personality and personality pathology to examine momentary and idiographic patterns of alliance rupture. Front Psychol. (2021) 12:711109. doi: 10.3389/fpsyg.2021.711109 34484067 PMC8415308

[80] Lobbestael J Arntz A Voncken M Potegal M . Responses to dominance challenge are a function of psychopathy level: A multimethod study. Pers disorders: Theory Res Treat. (2018) 9:305–14. doi: 10.1037/per0000252 28627902

[81] Voss C Raz T . Never Split the Difference: Negotiating as If Your Life Depended on it. New York, New York: Harper Business (2016).

[82] Choi-Kain LW Simonsen S Euler S . A mentalizing approach for narcissistic personality disorder: moving from "Me-mode" to "We-mode. Am J Psychother. (2022) 75:38–43. doi: 10.1176/appi.psychotherapy.20210017 35016552

[83] Asgarizadeh A Ghanbari S . Shame in borderline, antisocial and narcissistic personality disorders: Theoretical conceptualizations and empirical findings. Shenakht J Psychol Psychiatry. (2022) 9:1–15. doi: 10.32598/shenakht.9.5.1

[84] Dickinson KA Pincus AL . Interpersonal analysis of grandiose and vulnerable narcissism. J Pers Disord. (2003) 17:188–204. doi: 10.1521/pedi.17.3.188.22146 12839099

[85] Nathanson DL . The Many Faces of Shame. New York, New York: Guilford Press (1988).

[86] Pol SM Schug F Chakhssi F Westerhof GJ . Life stories of patients with personality disorders before and after treatment: Change and stability in agency and communion. Front Psychiatry. (2023) 14:1134796. doi: 10.3389/fpsyt.2023.1134796 37009106 PMC10060533

[87] Baltzersen Å . Statics to kinetics: Restoring the person to the center of personality disorder diagnosis. J Psychother integration. (2026) 36:147–9. doi: 10.1037/int000039427371692

[88] Gatner DT Douglas KS Almond MFE Hart SD Kropp PR . An economic analysis of crime costs associated with psychopathic personality disorder and violence risk. Criminal Justice Behav. (2023) 50:729–46. doi: 10.1037/per0000623

[89] Gullhaugen AS . (2012). Redefining psychopathy? Is there a need for a reformulation of the concept, assessment, and treatment of psychopathic traits? [Doctoral dissertation] NTNU (Norwegian University of Science and Thechnology). Available online at: https://ntnuopen.ntnu.no/ntnu-xmlui/handle/11250/270806 (Accessed October 14, 2026).

[90] Ribeiro da Silva D . (2026). Toward a suffering-informed framework of antisocial youth trajectories: Preliminary mixed-method evidence on compassion-flow configurations. Psychology, Crime & Law. dvance online publication. doi: 10.1080/1068316X.2026.2713582

